# Ferrochelatase: Mapping the Intersection of Iron and Porphyrin Metabolism in the Mitochondria

**DOI:** 10.3389/fcell.2022.894591

**Published:** 2022-05-12

**Authors:** Chibuike David Obi, Tawhid Bhuiyan, Harry A. Dailey, Amy E. Medlock

**Affiliations:** ^1^ Department of Biochemistry and Molecular Biology, University of Georgia, Athens, GA, United States; ^2^ Department of Microbiology, University of Georgia, Athens, GA, United States; ^3^ Augusta University/University of Georgia Medical Partnership, University of Georgia, Athens, GA, United States

**Keywords:** ferrochelatase, heme, iron, porphyrin, metabolon, porphyria

## Abstract

Porphyrin and iron are ubiquitous and essential for sustaining life in virtually all living organisms. Unlike iron, which exists in many forms, porphyrin macrocycles are mostly functional as metal complexes. The iron-containing porphyrin, heme, serves as a prosthetic group in a wide array of metabolic pathways; including respiratory cytochromes, hemoglobin, cytochrome P450s, catalases, and other hemoproteins. Despite playing crucial roles in many biological processes, heme, iron, and porphyrin intermediates are potentially cytotoxic. Thus, the intersection of porphyrin and iron metabolism at heme synthesis, and intracellular trafficking of heme and its porphyrin precursors are tightly regulated processes. In this review, we discuss recent advances in understanding the physiological dynamics of eukaryotic ferrochelatase, a mitochondrially localized metalloenzyme. Ferrochelatase catalyzes the terminal step of heme biosynthesis, the insertion of ferrous iron into protoporphyrin IX to produce heme. In most eukaryotes, except plants, ferrochelatase is localized to the mitochondrial matrix, where substrates are delivered and heme is synthesized for trafficking to multiple cellular locales. Herein, we delve into the structural and functional features of ferrochelatase, as well as its metabolic regulation in the mitochondria. We discuss the regulation of ferrochelatase via post-translational modifications, transportation of substrates and product across the mitochondrial membrane, protein-protein interactions, inhibition by small-molecule inhibitors, and ferrochelatase in protozoal parasites. Overall, this review presents insight on mitochondrial heme homeostasis from the perspective of ferrochelatase.

## 1 Introduction

The enzymatic chelation of a redox active metal into a small organic macrocycle, such as a porphyrin or corrin to produce cofactors including heme, chlorophyll, vitamin B12 and cofactor F430, represents one of the major benchmarks in evolution of cellular chemistry. In the instance of iron in heme, Nature has taken a highly reactive metal ion whose solubility near neutral pH is dependent upon redox state, and converts it into a metal chelated macrocycle whose solubility is independent of its redox state. In addition, the redox couple potential can be effectively optimized for diverse reactions by minor alterations in ring substituents, surrounding protein/solvent milieu, ring distortion, and/or presence of an axial ligand. The metalated macrocycle is facile in the types of reactions in which it participates and the types of ligands with which it may interact.

Metalated macrocycles and their associated metal chelatases are widespread in nature. Chelatases are grouped into three broad classes. The two largest groups are the Class I chelatases, which are composed of ATP-dependent heteromeric complexes, and the Class II chelatases, which are composed of ATP-independent monomeric or homodimeric proteins. Class I includes Mg chelatases for both chlorophyll and bacteriochlorophyll (BchlD, H, I), aerobic cobalamin biosynthetic cobalt chelatases (CobN, S, T), and a recently characterized nickel chelatase for cofactor F430 (Zheng et al., 2016; Moore et al., 2017). Members of Class II are the sirohydrochlorin ferrochelatase (SirB), the anaerobic cobalamin biosynthetic cobalt chelatases (CbiK and CbiX), and protoporphyrin IX and coproporphyrin III ferrochelatases. A third smaller class of chelatases, Class III, is composed of multifunctional dehydrogenases that also possess iron chelation ability. To date Class III chelatases are known only for siroheme biosynthesis (CysG and Met8p).

**FIGURE 1:**
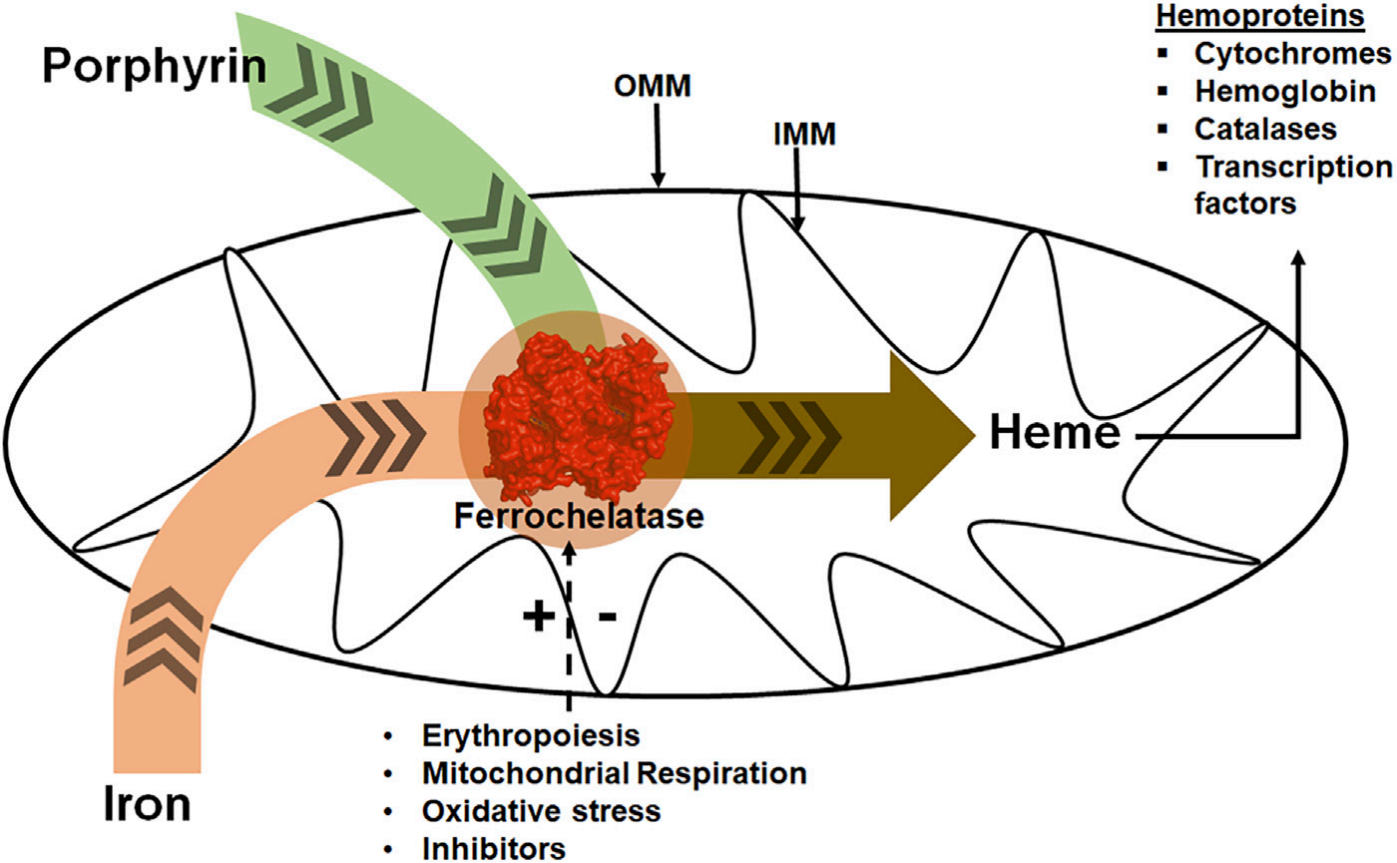
Ferrochelatase at the intersection of iron and porphyrin metabolism in mitochondria. Ferrochelatase (colored red) in the mitochondrial matrix catalyzes the insertion of ferrous iron into protoporphyrin IX to produce heme. Synthesized heme is then trafficked to hemoproteins. Physiological conditions such as erythropoiesis, mitochondrial respiration, and oxidative stress regulate this process. (+) and (−) signs represent stimulation or inhibition of ferrochelatase activity by these physiological conditions and dashed arrow represents regulatory process. IMM refers to the inner mitochondrial membrane and OMM to the outer mitochondrial membrane.

Examination and comparison of various chelatases along with nonprotein DNA- and RNAzymes and theoretical models for metal chelation have provided considerable insight and occasional confusion. The Class II chelatases are generally deemed as more simple enzymes given their size and lack of heteromeric structure. However, as detailed below, even these simple proteins utilize relatively complex methods to achieve their goal. Aside from CbiXs types of chelatases, Class II chelatase proteins possess a basic two-fold structural symmetry (Al-Karadaghi et al., 1997; Schubert et al., 1999; Wu et al., 2001; Karlberg et al., 2002; Romao et al., 2011) such that it is imaginable that the intact protein is the result of a gene fusion event with subsequent specialization. This was first proposed by Al-Karadaghi et al. (1997) who noted that the structure of the *Bacillus subtilis* ferrochelatase possessed two highly similar domains that could “have originated from a common ancestral protein”, an hypothesis that was supported by later structural studies on CbiXs of *Archaeoglobus fulgidus* (Yin et al., 2006). Much of our knowledge of Class II chelatases is from structural, enzymatic and mutagenesis studies of the ferrochelatases which function for heme biosynthesis. Ferrochelatases either utilize protoporphyrin IX or coproporphyrin III as one substrate and ferrous iron as the other substrate (Figure 1).

The idea that the insertion of iron to make heme occurred enzymatically originates from studies in the mid-1950s. Near simultaneous publications on characterization of iron insertion into protoporphyrin by cell-free extracts of avian reticulocytes came from Goldberg et al. (1956) and Klein et al. (1956). The proposed enzyme that catalyzes this biological process was originally called either heme synthase or protoheme ferrolyase (E.C. 4.99.1.1), but subsequently given the common name of ferrochelatase. Over the next two decades numerous studies from several laboratories reported on the presence of ferrochelatase activity and kinetic parameters for crude, cell-free extracts of the enzyme from a variety of bacterial and animal cell types. While there was general acceptance that the metalation reaction was enzyme catalyzed, two publications suggested that metalation may occur non-enzymatically (Tokunaga and Sano, 1972; Kassner and Walchak, 1973). This was resolved by the isolation and characterization of a mutant of the bacterium *Aquaspirillum itersonii* that lacked ferrochelatase activity *in vitro* and was a heme auxotroph (Dailey and Lascelles, 1974). A few years later, a yeast ferrochelatase-deficient mutant was also characterized (Gollub et al., 1977).

Until recently it was assumed that all ferrochelatases utilized the same porphyrin substrate, protoporphyrin IX. This assumption persisted in the face of confounding data including lack of rescue of some Gram-positive bacterial mutants with ferrochelatases from Gram-negative organisms or eukaryotes or very low measurable activity *in vitro* with protoporphyrin. Additionally, prior studies found that upon treatment with 5-aminolevulinic acid (ALA), Gram-negative bacteria including *Escherichia coli* and *Pseudomonas denitrificans* accumulated protoporphyrin, while the Gram-positive bacterium, *Staphylococcus aureus* and *Micrococcus lysodeikticus* accrued coproporphyrin (Townsley and Neilands, 1957; Jacobs et al., 1971). This remained an anomaly until 2015, when Dailey et al. (2015) identified a novel pathway in some Gram-positive organisms in which coproporphyrin ferrochelatase (CpfC previously known as HemH) catalyzes the penultimate reaction where ferrous iron is inserted into coproporphyrin III, instead of the canonical protoporphyrin IX. The coproheme is then decarboxylated by coproheme decarboxylase (ChdC previously known as HemQ) to form protoheme. This coproporphyrin-dependent heme biosynthesis pathway is specific to some Gram-positive bacteria in the Firmicutes and Actinobacteria Phyla, while in proteobacteria and eukaryotes, only the protoporphyrin-dependent pathway is found. With this discovery, a new classification, protoporphyrin ferrochelatase (PpfC) and coproporphyrin ferrochelatase (CpfC), is used to discriminate ferrochelatases when substrate specificity is discussed (Dailey et al., 2017).

**FIGURE 2:**
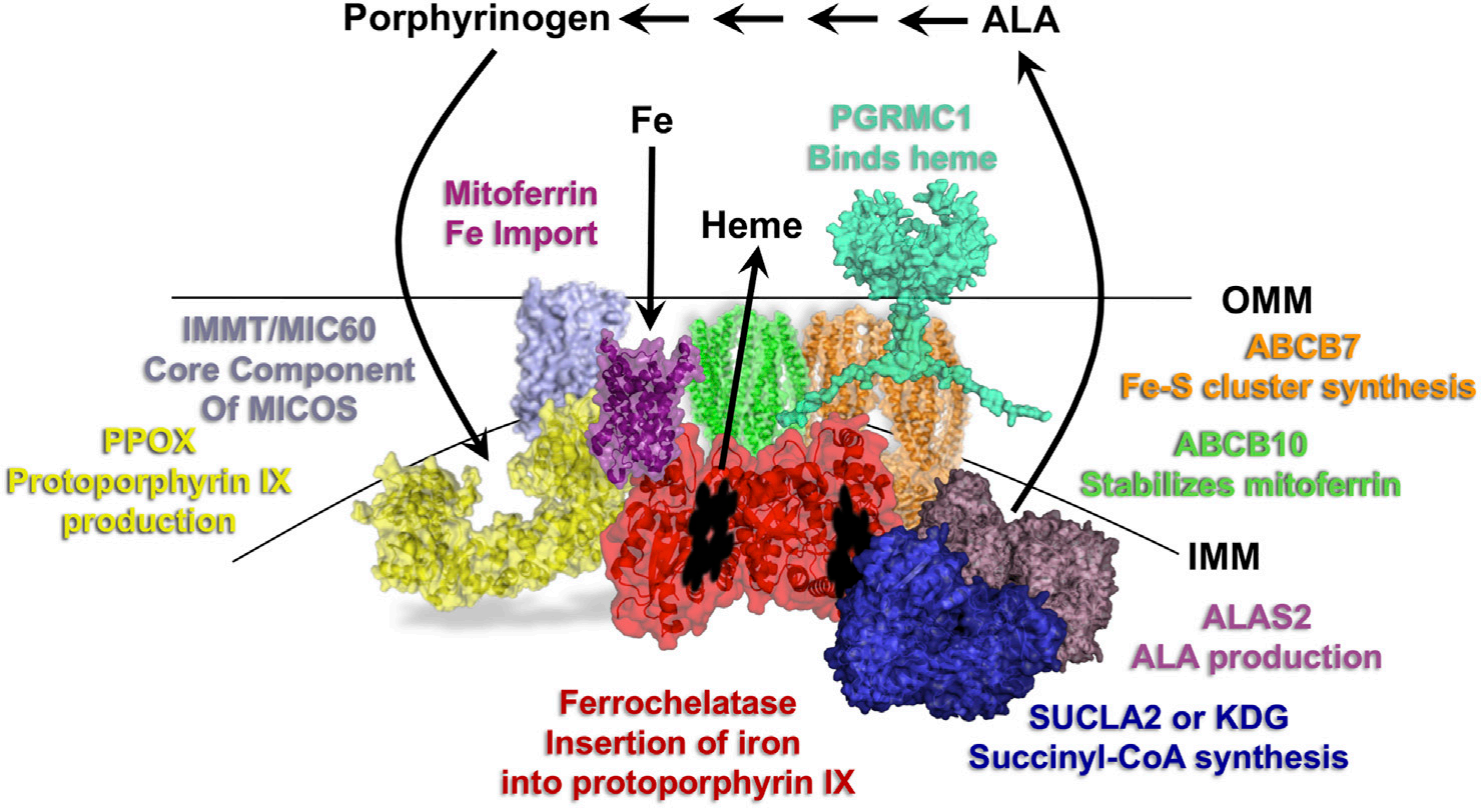
Overview of the heme synthesis pathway and model of key mitochondrial metabolon components. Localization of the first and last three reactions of the heme synthesis pathway occur in the mitochondria, with those in between occurring in the cytosol. Select proteins of the mitochondrial heme metabolon are shown and labeled with their roles in porphyrin, iron and heme homeostasis. IMM refers to the inner mitochondrial membrane and OMM to the outer mitochondrial membrane.

The current review focuses on metazoan and protozoan ferrochelatases, which all utilized protoporphyrin IX and ferrous iron and most are localized to the inside of the inner mitochondrial membrane (Figure 1). Plant ferrochelatases, for which there are two plastid-localized isoforms of ferrochelatase (FC1 and FC2), are not covered herein. In all other eukaryotes the first and last steps of the pathway occur in the mitochondria where enzymes of the heme synthesis pathway in the mitochondria exist in a multiprotein complex, or metabolon (Figure 2) (Medlock et al., 2015). The proteins of this metabolon are thought to facilitate substrate delivery, product release and regulation of heme synthesis. The complex roles of metabolon components are still being uncovered. Herein, we will review what is known about the metabolon components, substrate delivery and specificity, the [2Fe-2S] cofactor that many ferrochelatase possess, structure and kinetic-based catalytic mechanism, disease associated with ferrochelatase activity, and regulation of ferrochelatase activity through either protein-protein interactions, post-translational modifications, or inhibitors. We will also discuss the function of ferrochelatase in protozoal parasites and the direction of future ferrochelatase studies in the field.

## 2 Ferrochelatase Structure and Catalytic Mechanism

Over the past decades, the most significant advances in understanding the ferrochelatase mechanism have come from the availability of crystallographic data. First published in 1997 was the structure of *B. subtilis* ferrochelatase (CpfC) without bound porphyrin or protoheme, the presumed substrate and product (Al-Karadaghi et al., 1997). Shortly thereafter, structures became available for this CpfC with bound *N*-methylmesoporphyrin (Lecerof et al., 2000), a tight-binding competitive inhibitor (Marks et al., 1985; Gamble et al., 2000). In 2001 the first structure of a eukaryotic, human, ferrochelatase (PpfC) without bound substrate or product was published (Wu et al., 2001). To date over three dozen structures of ferrochelatases have been published (Supplementary Tables 1, 2). The majority of these are from two organisms, *B. subtilis* (CpfC)) (Al-Karadaghi et al., 1997; Lecerof et al., 2000; Lecerof et al., 2003; Shipovskov et al., 2005; Hansson et al., 2007; Karlberg et al., 2008; Hansson et al., 2011) and human (PpfC) (Wu et al., 2001; Medlock A. et al., 2007; Medlock A. E. et al., 2007; Dailey et al., 2007; Medlock et al., 2009; Medlock et al., 2012; Medlock et al., 2021). These structures are either of wild-type or variant forms of the enzyme some with substrate, inhibitor or product bound. In addition to the structures from these two species there are also structures from *Saccharomyces cerevisiae* (PpfC) (Karlberg et al., 2002; Dietz et al., 2021), *Listeria monocytogenes* (CpfC) (Hofbauer et al., 2020), and *Bacillus anthracis* (CpfC) (unpublished PDB ID 2C8J). No structures are available for any plant ferrochelatases. The current review is focused on the eukaryotic ferrochelatase, which are PpfC henceforth referred to as ferrochelatase, although information from structural studies of prokaryotic CpfC will be included as the mechanism of chelation is likely identical.

**FIGURE 3:**
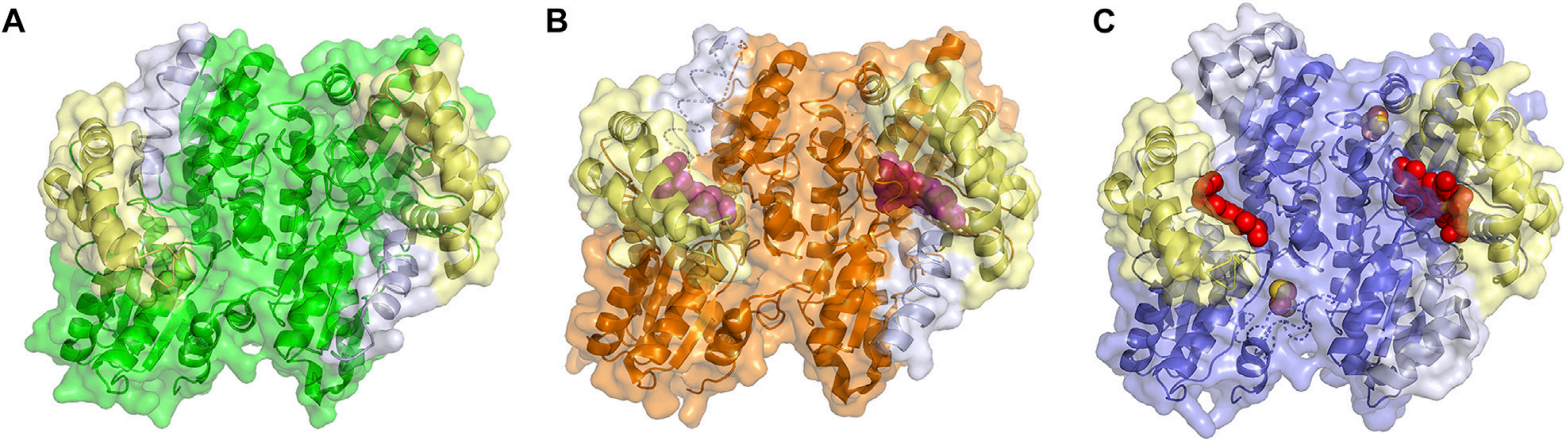
Structure of Ferrchelatase in open, closed and release conformations. Surface and cartoon representations of the **(A)** open conformation, shown in green (PDB ID 2HRC), **(B)** closed conformation, shown in orange (PDB ID 2QD1), and **(C)** release conformation, shown in blue (PDB ID 2QD2). The Fe-S clusters, shown in orange and yellow, in all structures as well as the bound porphyrin, shown in purple, in the closed conformation and heme, shown in red, in the release conformation are shown as spheres. The residues of the lip region are shown are colored pale yellow and π helix are light gray. Created using PyMOL Molecular Graphics System (Schrodinger, LLC).

When comparing ferrochelatase from human and *S. cerevisiae* with CpfC from *B. subtilis* there exist significant differences in the functional form, cellular location, and cofactor requirement. The human and *S. cerevisiae* enzymes function as homodimers (Burden et al., 1999; Wu et al., 2001; Grzybowska et al., 2002; Najahi-Missaoui and Dailey, 2005) and exist as a monotypic protein in the inner surface of the inner mitochondrial membrane (IMM) (Harbin and Dailey, 1985), while the *B. subtilis* CpfC functions as a soluble monomer (Hansson and Hederstedt, 1994). Human ferrochelatase possesses a [2Fe-2S] cluster (Dailey et al., 1994a; Dailey et al., 1994b) in each subunit coordinated by one internal cysteine and three cysteines that are located within the C-Terminus (Crouse et al., 1996; Sellers et al., 1998b). This cluster has a relatively low mid-point potential of approximately −450 mV (Burden et al., 1999). Interestingly the *S. cerevisiae* ferrochelatase lacks this cofactor (Dailey et al., 1994a; Hansson and Hederstedt, 1994), yet is similar in length to the human enzyme. When comparing the ferrochelatase from human with that of the CpfC, the human enzyme contains a 12 residue insertion in one “lip” of the active site pocket which is missing on the Gram-positive CpfC. This lip closes over the vinyl containing rings of the protoporphyrin IX macrocycle during catalysis and its absence in the CpfC is what allows those enzymes to bind coproporphyrin with its additional two propionate side chains (Dailey et al., 2015). With these differences and the low sequence homology between human ferrochelatase and *B. subtilis* CpfC (<10%), it is noteworthy that the overall three dimensional structures of each of the ferrochelatases solved to date are similar. The *B. subtilis* CpfC aligns with residues 130 to 390 of the human ferrochelatase monomer with an R.M.S.D. of 2.4 Å (cα′s). The overall structure of the human ferrochelatase monomer or *B. subtilis* CpfC is composed of two Rossmann-type domains which interact to form the active site (Al-Karadaghi et al., 1997; Wu et al., 2001). In addition all structures of ferrochelatases possess a π helix (Wu et al., 2001; Fodje and Al-Karadaghi, 2002) which contains several conserved residues (Figure 3) (Sellers et al., 2001).

The extension of structure to function has not been without some difficulties. All studies prior to 2015 assumed that all ferrochelatases used the same porphyrin substrate *in vivo*. This led to assumptions and extrapolations that have since lead to confusion. An example is that it was assumed that the spatial position of the *N*-methylmesoporphyrin as was seen in the *B. subtilis* CpfC was representative of the position of bound porphyrin substrate and product in the active site of all ferrochelatases during catalysis. As later demonstrated by crystallographic studies on human ferrochelatase with bound porphyrin substrate or product (Medlock A. et al., 2007; Medlock A. E. et al., 2007), both the position and orientation of protoporphyrin macrocycle in the active site pocket of the human enzyme is significantly different from that observed for *N*-methylmesoporphyrin bound to CpfC. Thus, previous interpretations of data prior to 2015 need to be reevaluated with this porphyrin substrate difference considered. Additionally, a number of early resonance Raman spectroscopic and mutagenesis studies on mouse ferrochelatase proposed involvement of some residues in the catalytic mechanism (Franco et al., 2000; Lu et al., 2002) that were later shown to be in error.

### 2.1 Impact of Substrate and Product Binding on Ferrochelatase Structure

What has been most useful to our understanding of how ferrochelatases function comes from the comparison of structures of wild type and variant human ferrochelatases with and without bound substrate or product. The structure of the human enzyme has been solved in three distinct conformations proposed to relate to steps during catalysis. These structures have been named open, which possesses no bound substrate or product; closed, which possesses bound porphyrin; and release, which has bound metalated porphyrin (product) (Figure 3) (Medlock et al., 2009). The proposed order in which the enzyme would proceed through these conformations during a complete catalytic cycle is open to closed to release, then returning to open for a new cycle. In the closed and release conformation the lip regions and π helix, respectively, are found in altered locations and orientations when compared to the wild-type enzyme in the open conformation (Supplementary Movie 1). It is of note that the closed and release conformations have not been observed in any ferrochelatase structures from other organisms which may be due to crystallization conditions and inhibitors utilized in these studies. To date only the open and partially closed conformations, between that of the open and closed conformations, have also been observed in the structure of the *S. cerevisiae* enzyme (Karlberg et al., 2002; Dietz et al., 2021).

### 2.2 Porphyrin Binding

**FIGURE 4:**
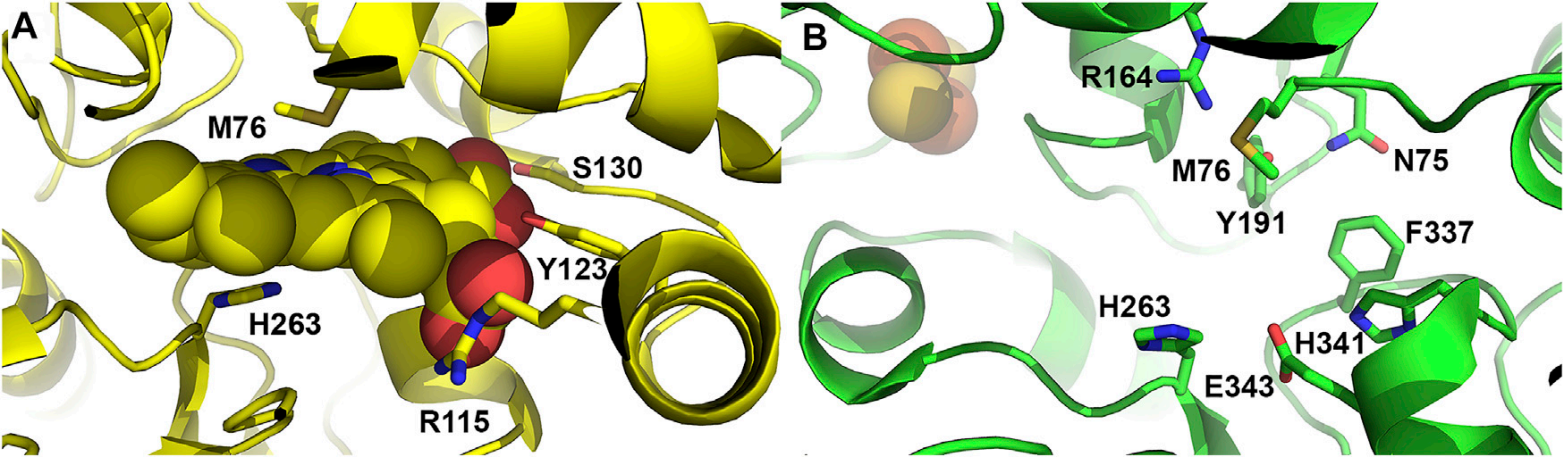
Active site residues of ferrochelatase. **(A)** Cartoon representation of the human ferrochelatase active site with protoporphyrin IX bound, shown as spheres, in the closed conformation. Key residues which interact with the porphyrin substrate and participate in catalysis are shown as sticks and labeled. **(B)** Cartoon representation of human ferrochelatase with key residues which participate in the hydrogen bonding network at the top and bottom of the active site shown as sticks and labelled. Created using PyMOL Molecular Graphics System (Schrodinger, LLC), PDB ID 2HRC **(A)** and PDB ID 2QD1 **(B)**.

Studies from 1979 by Honeybourne et al. (1979), provided information on the porphyrin substrate specificity of ferrochelatase. These studies showed that propionates are necessary at positions 6 and 7 of the tetrapyrrole and that other substituents were hydrophobic. Not until 2007 when the structure of human ferrochelatase with porphyrin bound was solved were the details of the molecular interactions uncovered. The human variant E343K was initially noted to be catalytically inactive and co-purify with large amounts of protoporphyrin bound (Sellers et al., 2001). The structure of the E343K (human numbering is used henceforth) variant was solved to reveal bound protoporphyrin IX in the active site. Furthermore, two additional protoporphyrin IX molecules were found just outside the entrance of the active site for several of the monomers suggesting a potential entry path for protoporphyrin IX into ferrochelatase. Within the active site several active site residues were found to interact with the ring substituents, specifically S130 and Y123 which hydrogen bond with propionate 6 and R115 which forms a salt bridge with propionate 7 (Figure 4A) (Medlock A. et al., 2007). Importantly, the overall structure of the protoporphyrin bound form was the first to demonstrate the dynamic nature of the enzyme, revealing that when porphyrin substrate was bound the active site mouth closed to completely engulf the macrocycle. This contradicted previous structural studies of the *B. subtilis* CpfC with *N*-methylmesoporphyrin bound (Shipovskov et al., 2005), which suggested ferrochelatases were rigid and static during the catalytic cycle. The human ferrochelatase structural data instead showed that a modest distortion of the porphyrin, ∼10°, is the mechanisms by which the enzyme carries out iron insertion (Medlock A. et al., 2007) which is consistent with resonance Raman studies (Blackwood et al., 1997).

### 2.3 Metal Binding

While ferrochelatase catalyzes the insertion of ferrous iron, a variety of other divalent cations, including nickel, cobalt, and zinc, may also serve as substrates *in vitro*. This low level of specificity is not of significance *in situ* since only iron and possibly zinc have access to ferrochelatase via transporters *in vivo*. Monovalent and trivalent cations are neither substrates nor inhibitors of ferrochelatase. The divalent heavy metals such as Mn, Hg, Cd, and Pb have long been considered to be inhibitors (Dailey, 1987), but recent studies show that the enzyme catalyzes the insertion of these metals. However, the metalated porphyrins created are poorly released by the enzyme (Medlock et al., 2009). Thus, the only metalated porphyrins produced *in vivo* are heme and zinc-protoporphyrin IX.

The pathway for movement of iron into the active site and its position within the active site of the enzyme are not currently experimentally defined. However, both structural studies (Medlock et al., 2012; Medlock et al., 2021). as well as molecular dynamics (Wang et al., 2013; Wang and Shen, 2013) and high-level quantum mechanical/molecular mechanics and quantum mechanical thermodynamic cycle perturbation (Wu et al., 2016) have provided significant insight. In the proposed model ferrous iron first undergoes desolvation either upon binding or during transit to the active site. The source of iron appears to be the mitochondrial solute carrier mitoferrin which has been shown to transport iron into the mitochondria for heme synthesis (Shaw et al., 2006) and interact with ferrochelatase (Chen et al., 2010). Models of a member of the mitochondrial solute carrier family with a known structure (SLC25A4) (Pebay-Peyroula et al., 2003) and ferrochelatase suggest that iron from mitoferrin would be directly donated without being released free into the matrix space. The iron would move via channel(s) in ferrochelatase to the active site for chelation. Channels which originate at the enzyme surface and terminate in the active site have been identified in human ferrochelatase (Medlock et al., 2012). When ferrochelatase variants at the protein surface near the channel which originates at surface residue H240 are introduced into ΔPpfC *S. cerevisiae* (previous ΔHem15), the complemented *S. cerevisiae* had decreased heme content even though the isolated variants have full enzymatic activity *in vitro*. Additionally, variants in which some of the H240 channel residues have been mutated showed altered kinetic parameters. These data support the role of the H240 channel in iron translocation from donation by mitoferrin at the back side of ferrochelatase to the active site pocket (Medlock et al., 2012). The protective channelling of iron from protein donors to the active site of ferrochelatase would be advantageous due to iron’s reactive nature that could cause oxidative damage to cellular components.

A potential regulatory metal binding site has been posited to exist for the mouse ferrochelatase based upon kinetic studies of variant ferrochelatases (Hunter and Ferreira, 2010) and for the *B. subtilis* CpfC from kinetic and crystallographic studies (Lecerof et al., 2003). Interestingly, some structures of the human and yeast ferrochelatases show a bound cobalt atom at the surface (Wu et al., 2001; Karlberg et al., 2002). Overall, given the complexity and high level of regulation of the iron supply pathway in metazoa and metal homeostasis in general, it is not clear what physiological role such a site might play and why cobalt or magnesium should function in a regulatory role for ferrochelatase.

### 2.4 Structure Supported Catalytic Model

The catalytic cycle begins with acquisition of one or both substrates by ferrochelatase in the open conformation. In this form the active site mouth is fully open and protoporphyrin IX could enter directly from protoporphyrinogen oxidase, the penultimate and prior enzyme in the heme biosynthetic pathway. Upon binding of porphyrin substrate the enzyme moves from the open to the closed conformation. In the closed form of the enzyme, the bound porphyrin is completely engulfed and sequestered from bulk solvent (Medlock A. et al., 2007). As described above structural and kinetics data support the movement of iron through a channel in the enzyme from the iron donor to the active site and binding at the M76 in the active site pocket. The order in which these substrate bind the enzyme is still unclear and it is of note that no structure of the human enzyme is currently available with only iron bound either in transit to or in the active site. Structural and recent kinetic studies are consistent with binding of the porphyrin substrate followed by iron (Sellers et al., 2001; Medlock A. et al., 2007) although older kinetic studies proposed metal binding prior to porphyrin (Dailey and Fleming, 1983).

The three identified structural conformations of the human enzyme along with structures from different species provide several clues to the impetus and mechanism to generate the closed conformation. Overall there is a concerted effort involving a number of active site-located residues (Medlock et al., 2021). As mentioned previously interactions of hydrophobic residues of the lip regions and the porphyrin macrocycle may play a role in the closing of the active site mouth. Additional interactions between the enzyme and the porphyrin propionate likely also contribute. Residues in the active site pocket which interact with the propionates of the porphyrin substrate are among the few identical residues found among all known ferrochelatases (Dailey et al., 2000). These include Y123 and S130 which are found within hydrogen bonding distance to the 6 propionate and R115 found within hydrogen bonding distance to the 7 propionate of protoporphyrin IX (Figure 4A). Of particular interest is residue S130 which is pointed towards the active site. In the human and *S. cerevisiae* structure residues G129, S130 and P131 are part of the loop connecting helix α3 and α4. Considering the location of S130 in the loop region between these two secondary structural elements and the level of conservation, this residue may play a role in mouth closing in addition to its role in binding of the porphyrin substrate. In the current model of human ferrochelatase in the inner mitochondrial membrane, S130 would exist near the interface of the inner mitochondrial membrane and the mitochondrial matrix. Interactions of proteins with ferrochelatase for substrate iron delivery or heme export could alter the orientation of this residue contributing the change in conformation observed in the (closed) substrate and (release) product bound structures. Interestingly, S130 has also been reported to undergo phosphorylation (Sakaino et al., 2009) which could alter substrate interaction.

In addition to the overall conformational change observed upon porphyrin binding, there is considerable remodeling of a number of active site residues. The active site of ferrochelatase contains the majority of the conserved amino acid residues and many of these conserved residues were found to participate in an extended hydrogen bond network which exists both above and below the porphyrin macrocycle (Medlock et al., 2021). In the open conformation, H263, E343 and H341 are found to participate in a hydrogen bond network at the bottom of the active site. At the top of the active site pocket residues M76, R164, Y191 and N75 are involved in a hydrogen bond network (Figure 4B) (Dailey et al., 2007; Medlock et al., 2012). While these networks are not connected by hydrogen bonds, there is some communication between them likely via the invariant F337 found at the back of the active site pocket. This residue, while conveying alterations in the hydrogen bond network, also plays a role in gating the previously mentioned tunnels which likely serve as conduits for iron, water or protons (Medlock et al., 2012).

Multiple studies have focused on residues H263, E343 and H341 as possible participants in metalation and/or deprotonation of the porphyrin ring nitrogens. The role of the invariant residue H263 in the catalytic cycle is well established via kinetic and structural studies on the enzyme from multiple organisms. All variants of H263 studied to date are inactive, including H263C as well as H263A in the presence of imidazole (Sellers et al., 2001; Dailey et al., 2007). Although H263 has been proposed to function both in metal binding (Kohno et al., 1994) and proton abstraction (Sellers et al., 2001), studies with catalytic antibodies (Cochran and Schultz, 1990) and nucleic acids (Conn et al., 1996; Li and Sen, 1996; 1997) as well as amide hydrogen/deuterium mass spectrometry (Asuru and Busenlehner, 2011) and computational studies (Sigfridsson and Ryde, 2003; Wang et al., 2013; Wang and Shen, 2013; Wu et al., 2016) are consistent with proton abstraction and metal insertion occurring on opposite face of the tetrapyrrole. In addition to H263 being conserved among all ferrochelatases, it is also the only basic residue in the vicinity of the pyrroles to be deprotonated. The role of E343 and H341 are consistent with the establishment of a hydrogen bonding network for proton abstraction by H263. The conservative replacements of E343 in the E343D variant which only shortens the length of the side chain by one carbon-carbon bond length significantly impacts catalysis at a step following chelation (Hoggins et al., 2007). Recent studies on the E343D and F110A/E343D variants have provided structural information that complements the catalytic data (Medlock et al., 2021). It is of note that E343 and H341 are residues in the conserved π helix, thus proton abstraction by H263 would disrupt the resting state hydrogen bond network in the π helix and provide an impetus for its unwinding.

The role of residues in the hydrogen bonding network at the top of the active site are less clear though recent studies have shed some light on the function of M76. M76 in the human enzyme is found opposite H263, the catalytic histidine. M76 is not conserved in all ferrochelatases, but is conserved in eukaryotic ferrochelatases. Mutagenesis studies of M76 show that this residue is fairly tolerant of changes as most variants retain residual *in vivo* activity as evaluated by rescue of *E. coli* deficient in ferrochelatase. *In vitro* activity, though, was unmeasurable for most M76 variants (Medlock et al., 2021). These findings along with those of other mutagenesis studies of neighboring residues, including R164, Y191 and N75, suggest several possibilities for the role of residues at the top of the active site in metal binding pre-chelation (Sellers et al., 2001; Dailey et al., 2007). First metal binding in the active site is transient and might require the presence of the macrocycle. Second metal binding likely involves multiple residues. These ideas are consistent with the weak interactions needed for chelation as well as the difference in the metal specificity observed for some organisms *in vitro*. While these differences may have little physiological relevance since specific transporters would exist to deliver iron *in vivo*, they do occur and would explain the lack of side chain conservation observed opposite the catalytic histidine, H263.

With the data from structural studies, as well as *in vitro* and *in vivo* analysis of ferrochelatase variants, the current model for the mechanism of ferrochelatases is as follows. First, porphyrin binds in the active site. Interactions between the porphyrin and enzyme trigger closing of the active site to completely engulf and cause minor distortion of the macrocycle. Second, or simultaneously, iron enters the active site via a surfaced originating channel and binds in the active site, opposite H263, at residues at the top of the active site pocket including M76, Y191 and N75. Third, two protons are abstracted via the hydrogen bonding network. The first proton could be lost to the bulk solvent while the second is removed by H263 which exists in the active site hydrogen bonding network making it a stronger base. Next, the protonated H263 would disrupt the hydrogen bond network causing movement of E343 and H341 and, thus, destabilize the π helix. In addition, metalation of the porphyrin in the active site of ferrochelatase also causes movement of a metal sensitive loop composed of residues Q302–Q314 (Medlock et al., 2009; Medlock et al., 2021). The movement of this metal sensitive loop likely further contributes to the unwinding of the π helix and together they lead to the formation of the release conformation (Medlock et al., 2021). In the release conformation, the partial unwinding of the conserved π helix at its N-terminus (residues 340–349) (Medlock A. E. et al., 2007; Medlock et al., 2009) results in large structural change for product release consistent with transient state kinetic studies which demonstrate that the slowest step in the catalytic cycle occurs following chelation (Hoggins et al., 2007). The unwinding of the π helix changes both the surface topology and the electrostatics surrounding the active site mouth thereby creating an alternate protein surface and a route for heme exit that is distinct from porphyrin entry (Medlock A. E. et al., 2007). This overall change in the surface structure and charge at the site of heme release functions to maximize interactions of ferrochelatase with heme chaperone(s) and/or transporter(s) so that they only occur when heme is available for transport. Last, for completion of the catalytic cycle, the enzyme would return to its original conformation, the open form. This would require rewinding of the π helix which probably occurs as heme leaves the active site by interactions with heme chaperone(s) and/or transporter(s).

## 3 Regulation of Ferrochelatase Activity

With advancements in science uncovering the intricacies of molecular and cellular biology, it has been widely established that *in vivo* activity of proteins is not a “hitch-free” process. In essence, many factors can positively or negatively affect enzyme activities post translationally. Like many enzymes, ferrochelatase activity can be regulated through covalent and non-covalent modifications. These modifications may induce changes in the enzyme’s structure or introduce additional molecular features as regulatory measures. Small molecules can also directly or indirectly regulate ferrochelatase activities through inhibition and feedback regulations. These factors offer organisms the leeway to dynamically respond to heme-associated stimuli or adjust to different cellular heme demands. Here, we review different means by which ferrochelatase activity is or could be regulated post translationally.

### 3.1 The Mitochondrial Heme Metabolon

Although proposed earlier (Grandchamp et al., 1978) it was not until 1988 that the first experimental data were presented in support of the hypothesis that ferrochelatase interacted with protoporphyrinogen oxidase (PPOX) *in situ* (Ferreira et al., 1988). Later this was expanded to demonstrate experimentally that an interaction involving ferrochelatase, PPOX, and coproporphyrinogen oxidase (CPOX) occurs but that it is probably of a transient nature (Proulx et al., 1993). With the availability of crystal structures for eukaryotic ferrochelatase and PPOX, in silico docking experiments provided structure-based support for this model (Koch et al., 2004). In addition, it has been shown that ferrochelatase interacts with the mitochondrial inner membrane iron transporter, mitoferrin and its partner ABCB10 (Chen et al., 2010; Medlock et al., 2015) and considerable experimental data demonstrate that mitoferrin may serve as the iron transporter *in vivo* for heme synthesis (Shaw et al., 2006). The proposal that the matrix-located molecule frataxin serves a role as an iron donor to ferrochelatase (Yoon and Cowan, 2004) seems unlikely given that in Friedreich’s ataxia, where frataxin is deficient, there is no evidence for diminished heme synthesis (Williams and De Jesus, 2022).

More recent co-immunoprecipitation (co-IP) studies using epitope-tagged human ferrochelatase in murine erythroleukemia cells have validated these interactions and uncovered additional proteins partners for ferrochelatse (Medlock et al., 2015; Piel et al., 2016). A surprising finding from these studies was that ferrochelatase interacts with the first enzyme in the heme biosynthetic pathway, erythroid 5-aminolevulinate synthase (ALSA2) (Figure 2). Additionally, ferrochelatase was also found to interact with the beta subunit of succinyl-CoA synthetase (SUCLA2) and α-ketoglutarate dehydrogenase (KDH). SUCLA2 is a known partner of ALAS2 (Bishop et al., 2012). KDG was more recently shown to interact with ALAS2. This interaction is the means by which erythroid cells provide the carbon source from glutamine for the huge heme demand required during erythroid differentiation (Burch et al., 2018). These interactions along with the finding that porphyrin precursors increase with elevations in ferrochelatase levels (Medlock et al., 2015) suggest that ferrochelatase plays a role in regulating heme synthesis via porphyrin precursor production and explain how the physiological demands for heme metabolism drives the establishment of the heme metabolon.

In addition to interacting with other mitochondrial proteins for substrate channelling, ferrochelatase may also interact with these proteins to enhance its stability *in vivo* or regulate mitochondrial iron homeostasis. ABCB7, a transporter involved in Fe-S cluster biogenesis, and ABCB10, which also interacts with mitoferrin, form a complex with ferrochelatase. Knock-down of ABCB7 in erythroid cells led to mitochondrial iron overload and defective heme biosynthesis (Maio et al., 2019). The stability of ferrochelatase was also reported to be lowered by ABCB7 depletion. Thus, this complex is not only essential for mitochondrial heme biosynthesis, but also crucial for iron and Fe-S cluster homeostasis.

As heme is synthesized in the mitochondrial matrix, it is expected to be chaperoned from ferrochelatase either for immediate use in mitochondrial cytochrome assembly or trafficked to other sites. Progesterone receptor membrane component 1 (PGRMC1) has been recently identified through its interaction with ferrochelatase as a potential mitochondrion-to-cytoplasmic heme chaperone candidate. Unlike glyceraldehyde-3-phosphate dehydrogenase which has been shown to be a cytoplasmic heme chaperone (Sweeny et al., 2018), PGRMC1 is a protein localized to the outer mitochondrial membrane (OMM) (Piel et al., 2016) with an N-terminal region that spans across the membrane. It has heme binding capability via a cytochrome b_5_-like heme binding motif (Mifsud and Bateman, 2002; Ghosh et al., 2005). PGRMC1 interacts most strongly with ferrochelatase in its heme bound/product release conformation (Piel et al., 2016). Also, it can efficiently donate its bound heme to some apo-hemoproteins, making it an ideal heme chaperone. However, the mechanism by which heme synthesized in the mitochondrial matrix is transferred to PGRMC1 in the OMM remains elusive. The formation of a transient or stable heme trafficking complex in the mitochondria likely exists to facilitate this process.

In mouse and *S. cerevisiae* cells, ferrochelatase has been shown to interact with components of the mitochondrial membrane contact site and cristae organizing system (MICOS). Of the eight known proteins that make up this complex, five were found to physically interact with *S. cerevisiae* ferrochelatase via co-IP (Dietz et al., 2021). Interestingly, mammalian homologs of MICOS have also been found to interact with human ferrochelatase (Piel et al., 2016). MICOS is essential for the formation of mitochondrial cristae structure. In doing so, it tethers the IMM to the OMM. Deletion of *Mic60*, one of the key components of MICOS that interact with *S. cerevisiae* ferrochelatase, leads to impaired respiratory growth and heme biosynthesis when *S. cerevisiae* ferrochelatase is overexpressed. Levels of heme intermediates were also altered. Protoporphyrin IX levels decreased while other porphyrin intermediates increased. Restabilizing this connection between the OMM and IMM via a synthetic tether rescued the phenotype (Dietz et al., 2021). In sum, the integrity of this mitochondrial interface is crucial for efficient ferrochelatase activity and heme biosynthesis.

### 3.2 Posttranslational Modification of Ferrochelatase

Post-translational modifications (PTMs) of proteins are a commonly observed feature in cells to modify protein specificity or activity, diversify their functions, and dynamically regulate cellular signaling networks (Deribe et al., 2010; Theillet et al., 2012). In eukaryotic cells phosphorylation, acetylation, ubiquitination, glycosylation, and glutathionylation, are some common modifications that are pivotal in regulating protein function, localization, and stability (Theillet et al., 2012; Lee and Yaffe, 2016). These covalent modifications induce conformational change(s) that alter their biological activities, and/or regulate their interactions with other proteins, cofactors, nucleic acids, or lipids (Lee and Yaffe, 2016). Proteins with several modification sites can undergo a hierarchy of PTMs in response to different stimuli. These modifications can be irreversible or reversible, thus enhancing the dynamics of cell signaling and homeostasis. Consequently, pathogenesis of several diseases has been associated with defects in PTMs (Gong et al., 2005; Morino et al., 2005; Jin and Zangar, 2009; Santos and Lindner, 2017; Xu et al., 2018).

Cellular demands for heme differ based on cell type, redox state, energy requirements, hormonal signals, hypoxia, and other physiological conditions. Thus, heme biosynthesis needs to be dynamic to meet these contrasting cellular requirements. Gene expression and regulation has been extensively studied to understand how cell transcriptome changes in response to heme demand or regulation (Phillips and Kushner, 2005; Nilsson et al., 2009). However, the possible effects of PTMs of ferrochelatase and other heme biosynthesis enzymes and chaperones have been explored less. PTMs allow for a rapid and reversible response to various environmental or developmental alterations and a more direct mechanism of physiological regulation of heme. Below we review PTMs of metazoan ferrochelatase.

#### 3.2.1 Phosphorylation

Protein kinases are known to catalyze the phosphorylation of proteins in an ATP-dependent manner. Studies have reported that ferrochelatase is targeted for phosphorylation by protein kinase C (PKC) and protein kinase A (PKA) (Sakaino et al., 2009; Chung et al., 2017). It was reported that ferrochelatase phosphorylation by PKA may be more physiologically relevant than PKC, as PKC-mediated ferrochelatase phosphorylation leads to no changes in the enzyme’s activity (Sakaino et al., 2009). However, care must be taken when extrapolating the potential physiological impact of modifications that do not alter *in vitro* enzyme activity since these surface residues modified by PKC may have an impact on *in situ* protein-protein interactions. In contrast to PKC, PKA regulatory and catalytic subunits are upregulated alongside other heme metabolism proteins during erythropoiesis (Nilsson et al., 2009; Chung et al., 2012; Chung et al., 2017). Erythropoietin (EPO), a hormone produced in the kidney and known to stimulate production of erythroid cells in the bone marrow, drives this process to elevate hemoglobin production (Tanaka and Nangaku, 2012; Chung et al., 2017). Upon binding to its receptor, EPO-R, in maturing erythrocytes, several kinases critical for proliferation and differentiation of erythroid progenitors are activated (Fibach, 2011; Chung et al., 2012). PKA upregulated at this stage is then localized to the outer mitochondrial membrane via interactions with an anchoring protein, AKAP10. Further investigations revealed that PKA phosphorylates residue T116 of ferrochelatase, in the α-helix lip that opens to the active site pocket, likely during translocation to the mitochondrial matrix (Chung et al., 2017).

**FIGURE 5:**
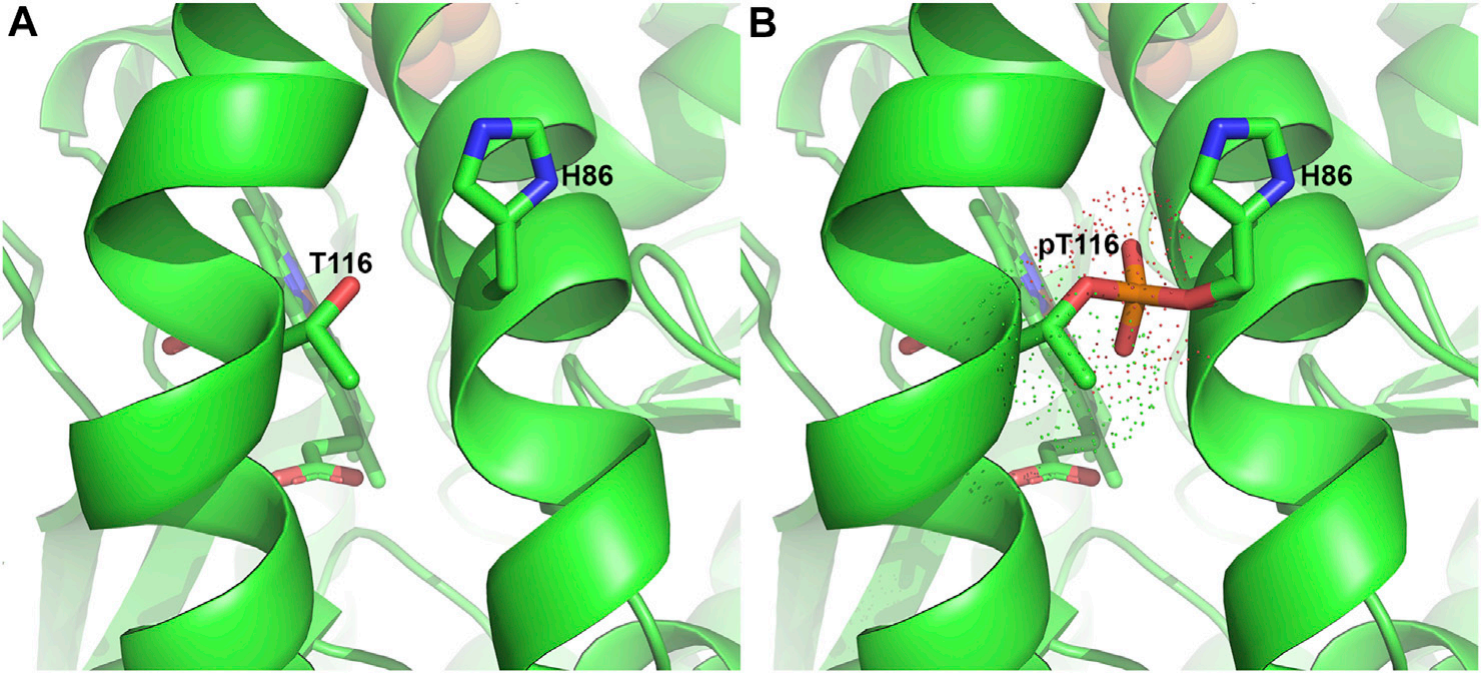
Phosphorylation of T116 in Ferrochelatase. **(A)** Cartoon representation of the human ferrochelatase (PDB ID 2QD2) without any modification at T116, shown as sticks and labeled. **(B)** T116 with covalently bound phosphate group, shown in orange, at T116 overlapping H86, shown as sticks and labeled. Dots represent the electron density of pT116. Modeled using Vienna-PTM (Margreitter et al., 2013) and figure created using PyMOL Molecular Graphics System (Schrodinger, LLC).

The phosphorylation of T116 has been proposed to induce a conformational change, moving the bulky phosphorylated T116 (pT116) away from H86 on another α-helix. Structural modeling with Vienna-PTM (Margreitter et al., 2013) shows that electron density of pT116 would overlap with H86 (Figure 5), thereby making this proposed conformational change plausible. Enzyme kinetic analysis of phosphorylated ferrochelatase showed an increase in activity (Chung et al., 2017), suggesting that the conformational change is significant to alter the enzyme catalysis. Since T116 is highly conserved in eukaryotic ferrochelatases, this PTM could be an indispensable process for metazoans especially during hemoglobinization. To buttress that, knock-out of AKAP10 and knock-in of the analogous T116A substitution into the endogenous ferrochelatase gene in separate occasions led to decreased ferrochelatase activity in murine erythroleukemia cells (Chung et al., 2017). Upon further characterization of phosphorylated ferrochelatase, we speculate there could be additional PKA-dependent phosphorylation sites. The potential physiological relevance of adding phosphate group at these alternate sites remains to be determined.

#### 3.2.2 Glutathionylation

Cysteine residues are well known as molecular redox switches (Klomsiri et al., 2011; Fra et al., 2017). They are frequently buried beneath the surface of proteins, but when surface-located they may be important for protein function or regulation. The cysteine thiol group can undergo several modifications when converted to its thiolate, a more reactive form. These modifications include *S*-glutathionylation, *S*-hydroxylation, *S*-nitrosylation and disulfide linkage (Chung et al., 2013). Conversion of thiol to thiolate is dependent on the physiological pH of the cell and reactive oxygen species (ROS) levels (Winterbourn and Hampton, 2008; Schieber and Chandel, 2014). Thus, cysteine residues have the potential to regulate cell signalling and maintain redox homeostasis by altering protein structure through these modifications, then, protein function or activity.

Glutathionylation is one of the most common modifications of cysteine residues. It is a redox-dependent process that involves attachment of glutathione (GSH) to these residues (Gallogly and Mieyal, 2007; Mailloux and Willmore, 2014). With mitochondria being the primary site for redox reactions and targets for oxidative damage by ROS, mitochondrial proteins may be susceptible to redox-related modifications. A heme biosynthetic enzyme, uroporphyrinogen decarboxylase, has been identified to undergo this PTM in *S. cerevisiae* (McDonagh et al., 2013). In addition, heme is essential for redox chemistry, as many proteins involved in this process use heme as their cofactor. These findings suggest that cellular redox state could regulate heme biosynthesis. In essence, ferrochelatase and other mitochondrial proteins that contain regulatory cysteine residues are targets of *S*-glutathionylation in response to shift in the redox state of the cells (McGarry et al., 2015). Human ferrochelatase has 9 cysteine residues with 2 of these being surface located. One surface-exposed cysteine residue, C395, is near the ferrochelatase [2Fe-2S] cluster binding site. [2Fe-2S] clusters have been reported as redox sensors, and data are consistent with such a function of the cluster in ferrochelatase (Shah et al., 2012). Nevertheless, the activity of ferrochelatase decreases in a concentration-dependent manner when exposed to nitric oxide (Sellers et al., 1996). Glutathionylation of cysteine residues in ferrochelatase might be of physiological relevance, hence, worth investigating. Furthermore, thioredoxin and glutaredoxin facilitate the exchange of GSH with cysteine residues (Gallogly and Mieyal, 2007; Winterbourn and Hampton, 2008) and have been reported to regulate porphyrin biosynthesis in plants and metazoans (Da et al., 2017; Daher et al., 2019). Mitochondrial glutaredoxins, especially GLXR5, are crucial for heme biosynthesis in eukaryotic cells (Wingert et al., 2005; Daher et al., 2019). Beyond trafficking Fe-S clusters, they can facilitate glutathionylation and deglutathionylation of target proteins. To this end, investigating the physiological relevance of ferrochelatase glutathionylation is key towards understanding how these redox molecular switches might influence heme biosynthesis.

#### 3.2.3 Acylation

Acylation of lysyl residues is also a common PTM. Ferrochelatase possesses a number of surface-located lysyl residues that are subject to succinylation in the presence of succinyl CoA. As expected, a global analysis of lysine succinylation in mouse liver identified ferrochelatase as a succinylation target (Weinert et al., 2013). The fact that ferrochelatase is located in the mitochondrial matrix where succinyl CoA is synthesized and utilized by ALAS for the first step of heme synthesis in metazoans, makes the possibility that this PTM is involved *in vivo* in regulation of heme synthesis by modification of ferrochelatase an attractive hypothesis. Of note is that like glutathionylation, acylation is sensitive to and regulated by cellular redox (Jones, 2010).

### 3.3 Ferrochelatase Inhibitors

#### 3.3.1 Endogenously Generated Inhibitor

In the 1980s De Matteis and collaborators discovered the formation of a “green” porphyrin analogue after treating mice with 3,5-dicarbethoxy-1,4-dihydrocollidine (DDC) (De Matteis et al., 1985). DDC and some of its 4-alkyl analogues were dubbed “suicide substrates” for cytochrome P450 since during catalytic turnover a portion of P450s inappropriately alkylate the prosthetic heme group at one of its pyrrole rings with a methyl group from DDC with the concomitant release of iron. This generated an *N*-alkylprotoporphyrin, *N*-methylprotoporphyrin, which in turn inhibits ferrochelatase *in situ*. However, not all *N*-alkylprotoporphyrins inhibit ferrochelatase. *N*-isobutylporphyrin generated from DDC 4-isobutyl analogue did not have ferrochelatase inhibitory activity. Griseofulvin and Cremastranone are anti-fungal drugs that can elicit photosensitivity (Kawabe et al., 1988) and also inhibit ferrochelatase *in vivo*. Griseofluvin, like DDC, causes a suicide P450 reaction to produce *N*-methylprotoporphyrin. It is assumed that Cremastranone functions in the same fashion. Griseofulvin has been studied in cell cultures as a retinal neovascularization agent (Basavarajappa et al., 2017; Pran Babu et al., 2020). Inhibition of ferrochelatase has been shown to promote physiological ocular angiogenesis and vascular repair suggesting that ferrochelatase may represent a druggable target in the treatment of this disease (Sishtla et al., 2022).

It was demonstrated by others that *N*-methylprotoporphyrin is a tight-binding competitive inhibitor of purified ferrochelatase with a nanomolar K_i_ (Dailey and Fleming, 1983). Marks and others examined a variety of *N*-alkylated porphyrins in the chick embryo system (Marks et al., 1985) and with purified ferrochelatase (Gamble et al., 2000) and demonstrated that both the nature of the alkyl group along with the particular pyrrole ring modified had a significant role in the effectiveness of inhibition. *N*-methylmesoporphyrin and *N*-methylprotoporphyrin have been extensively used in a number cellular and animal studies where inhibition of heme synthesis was sought. Unfortunately, many of these are of questionable value since they have been poorly controlled and, not surprisingly, lack proper citation. In cases where ferrochelatase may be deemed as a target for clinical intervention, care must be taken to ensure that in humans there are not potential photosensitivity and/or anemia as side effects.

#### 3.3.2 Protein Kinase Inhibitors

Some protein kinase inhibitors used as cancer chemotherapeutics, such as Alectinib, Cyc-116, Dasatinib, and Vemurafenib, are known to elicit photosensitivity as one of their side effects (Chapman et al., 2011; Peters et al., 2017; Tarantini et al., 2019). Further characterization of these drugs revealed that most of them bind to and inhibit ferrochelatase (Klaeger et al., 2016). Inhibition of ferrochelatase after administration of these drugs leads to accumulation of protoporphyrin IX. Protoporphyrin IX is a photosensitizer that is toxic to cells upon accumulation and light exposure. The symptoms are reminiscent of EPP symptoms, but not as severe. However, the phototoxicity side effect has been investigated for use in photodynamic therapy (PDT) treatment of some skin lesions from parasitic infections (Dai et al., 2009; Peloi et al., 2011; Chang et al., 2016), or cancers (Sibata et al., 2001; Dolmans et al., 2003; Agostinis et al., 2011; Fukuhara et al., 2013).

**FIGURE 6:**
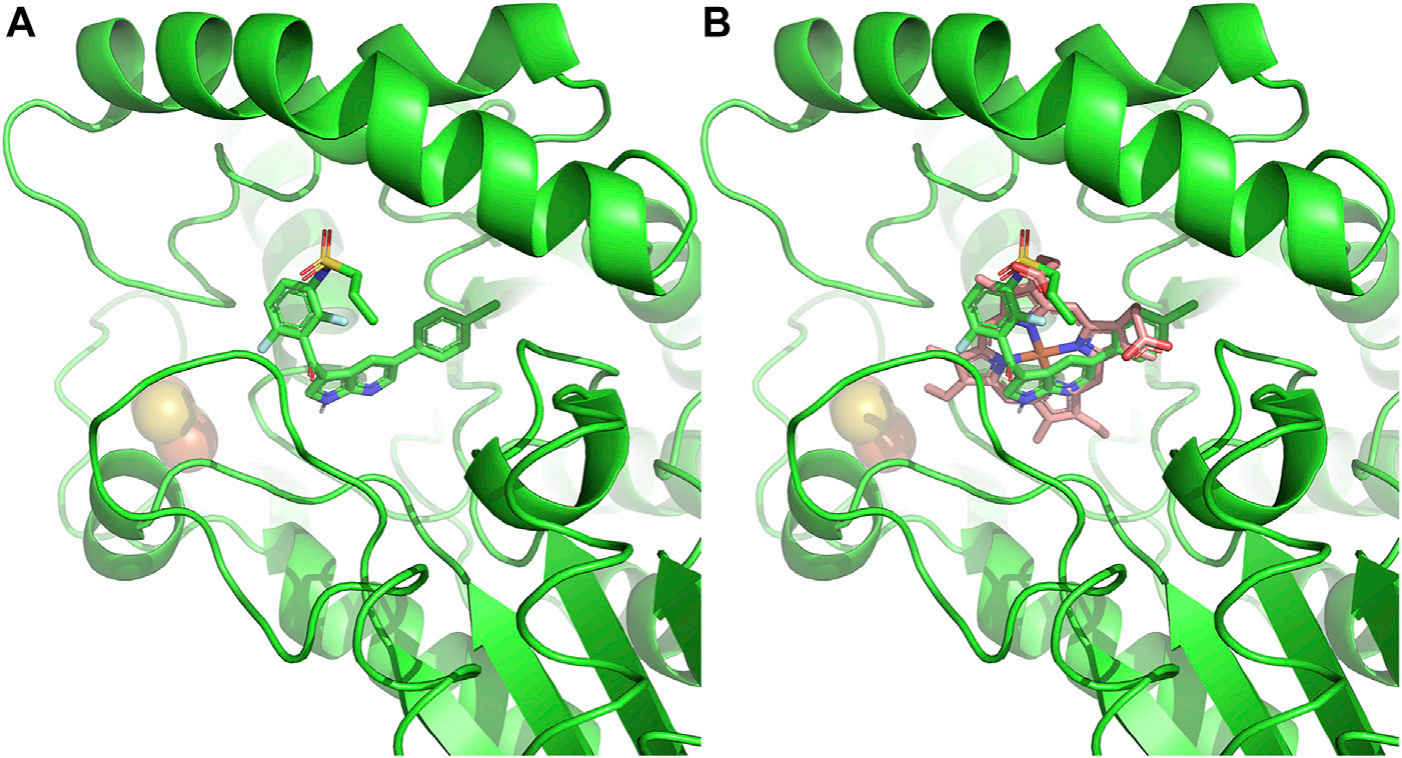
Ferrochelatase–Vemurafenib complex. **(A)** Cartoon representation of Vemurafenib bound to the active site of human ferrochelatase (PDB ID: 2QD3). **(B)** Vemurafenib bound to the active site of human ferrochelatase with heme. Molecular docking of was performed using AutoDock Vina (Trott and Olson, 2010) and figure created using PyMOL Molecular Graphics System (Schrodinger, LLC).

Beyond the phototoxicity effect of off-target ferrochelatase inhibition by these chemotherapies, cellular heme depletion could also be a concern. Recently, nephrotoxicity of B-RAF-kinase inhibitor, Vemurafenib, in patients with metastatic melanoma has been attributed to ferrochelatase inhibition (Bai et al., 2021) (Figure 6). Of note is that Dabrafenib, a B-RAF inhibitor that does not inhibit ferrochelatase (Klaeger et al., 2016), has less renal toxicity than Vemurafenib (Jhaveri et al., 2015). The renal toxicity of Vemurafenib is independent of light exposure, hence, not triggered by protoporphyrin IX accumulation. Studies suggest that it could be as a result of heme depletion instead, since knockdown of ferrochelatase accelerates the Vemurafenib-induced nephrotoxicity while ferrochelatase overexpression compensates for it (Bai et al., 2021). Thus, kinase inhibitors with off-target ferrochelatase inhibition might pose a risk of inducing mitochondrial dysfunction.

#### 3.3.3 Non-Kinase Inhibitors

In addition to kinase inhibitors, other medications have been characterized as ferrochelatase inhibitors. Salicylic acid, an Non-steroidal anti-inflammatory drugs (NSAID), has been reported to inhibit ferrochelatase activity by binding to the dimer interface in two possible orientations (Gupta et al., 2013). Structural and biochemical data from this study show that ferrochelatase residues W301 and L311 (human numbering) that are involved in binding to salicylic acid, are also essential for ferrochelatase dimerization. Mutation of these residues to alanine led to the formation of monomeric, inactive variants of ferrochelatase (Gupta et al., 2013). Several studies have shown that ferrochelatase is active as a homodimer (Burden et al., 1999; Wu et al., 2001; Grzybowska et al., 2002; Najahi-Missaoui and Dailey, 2005). Consequently, disrupting the dimerization of this enzyme would affect its activity. It is of note that some ferrochelatase-inhibiting kinase inhibitors have also been suggested to inhibit ferrochelatase activity by binding to its dimer interface (Klaeger et al., 2016).

Given these observations, it is logical to consider ferrochelatase inhibition for drug induced photosensitivity. Hence, drugs known to elicit photosensitivity such as quinine, lovastatin, ciprofloxacin, etc. are recommended to be studied for their effect on ferrochelatase activity. Moreover, while studying the molecular scaffold of off-target ferrochelatase inhibitors, we realized that most of them have a fused-heterocyclic ring with at least one aromatic center. Thus, we speculate that other compounds with similar structural features might as well be potential ferrochelatase inhibitors. Natural products such as Staurosporine and Rebeccamycin that possess these structural scaffolds should be considered as candidates. Identifying other pharmacophores that trigger ferrochelatase inhibition will help towards modifying existing off-target ferrochelatase inhibitors to minimize the effect or optimizing potential ferrochelatase inhibitors to increase potency. In summary, inhibition of ferrochelatase has been used to improve ALA-based PDT against prostate cancer (Fukuhara et al., 2013) and reduce choroidal neovascularization (Basavarajappa et al., 2017), a major cause of visual loss, highlighting the value of such inhibitors in the clinical field.

## 4 Disease Associated With Ferrochelatase

There are two recognized genetic disorders related to human ferrochelatase activity. In one, named erythropoietic protoporphyria (EPP), there is decreased activity of the enzyme (Dailey and Meissner, 2013). In the other, X-linked protoporphyria (XLP), ferrochelatase levels are normal, but there is a gain of function in the first pathway enzyme, ALAS2 which leads to the production of protoporphyrin that is in excess over what the levels of ferrochelatase normally present can metabolize (Whatley et al., 2008). Both EPP and XLP are erythroid forms of disorders that are categorized as “cutaneous” porphyrias since clinical manifestations are generally limited to photosensitivity (Phillips, 2019). The main distinction is that in EPP one finds accumulation of free protoporphyrin IX while in XLP there is significant accumulation of zinc-protoporphyrin IX (Di Pierro et al., 2022).

EPP, while classified as a rare disease, is one of the more common porphyrias affecting individuals worldwide. It is the most common porphyria in children, third most common in adults and of equal incidence between male and female (Balwani et al., 1993). In EPP one finds only about 30% of normal levels of ferrochelatase activity. This diminished activity generally results from one allele encoding a protein with a point, missense or deletion mutation that causes diminished enzyme activity (Brenner et al., 1992; Dailey et al., 1994b; Sellers et al., 1998a) and a second allele that may have a normal coding region for ferrochelatase, but has a mutation that causes low expression of that allele (Gouya et al., 1996). Affected individuals are heterozygous for both the mutated variant and a low functioning allele (IVS3-48T/C). Clinical disease is not present in those homozygous for IVS3-48T/C (Gouya et al., 1999; Gouya et al., 2002). EPP can also result from an autosomal recessive loss-of-function mutation in both ferrochelatase gene alleles (Lamoril et al., 1991; Sarkany et al., 1994).

In contrast in XLP, there are normal levels of ferrochelatase enzyme activity, but increased protoporphyrin production due to a gain of function mutation in ALAS2 (Whatley et al., 2008). The mutation involves C-terminus alterations which cause the elevation of ALAS2 activity by preventing this segment of the enzyme, which appears to serve a regulatory purpose, from properly folding over the active site of ALAS2 (Bailey et al., 2020). This results in the overproduction of downstream intermediates and protoporphyrin IX in particular. Another variant of the disorder results from a mutation to CLPX, a mitochondrial protease which degrades ALAS2. This leads to an increase of ALA and protoporphyrin (Whitman et al., 2018; Yien et al., 2017).

Clinical manifestations of EPP and XLP are consistent with other cutaneous porphyrias in that they can be nonspecific or difficult to diagnose (Balwani, 2019). These include burning, stinging and prickling. However, unlike other porphyria’s, post exposure pain can linger for minutes to days and leaves little to no scarring (Holme et al., 2006). This fact makes it difficult for clinicians to pinpoint a diagnosis leaving patients either undiagnosed or misdiagnosed. Patients can present in early childhood with symptoms of photosensitivity, typically under 4 years of age. Studies have revealed patients waiting approximately 13 years from presenting symptoms to diagnosis with 40% having consulted five physicians and 20% more than 10 before an ultimate diagnosis (Lala et al., 2018).

Painful non-blistering photosensitivity is the most common manifestation. Pain akin to placing affected areas over a flame have been described. Very rarely patients can present with severe symptoms including edema or petechiae. Overt signs of the disease come in the form of skin thickening or lichenification on the back of hands around the knuckles, as well as vertical grooving on the lips and loss of lunulae of fingernails (Schmidt et al., 1974; Baart de la Faille et al., 1991). EPP/XLP can also cause liver failure and gallstones (Todd, 1991; Lecha et al., 2009; Casanova-Gonzalez et al., 2010). Hence, EPP should be considered as a differential if gallstones are found in children, the presentation is otherwise typical. Liver disease in these patients, referred to as protoporphyric hepatopathy, is very rare but rapidly progressive (Bloomer, 1988). Peripheral neuropathy (in those with protoporphyric hepatopathy) (Rank et al., 1993), anemia, and vitamin D deficiency are also seen in EPP patients (Spelt et al., 2010; Allo et al., 2013).

Diagnostic evaluations in EPP/XLP patients are difficult due to reasons previously stated. There are many causes of photosensitivity, however the acute and non-blistering nature of EPP/XLP may illicit suspicion. Physicians may recognize hypochromic microcytic anemia on blood smear/RDW, as well as low ferritin concentration and transferrin saturation (Mittal and Anderson, 2022). An ultimate diagnosis can be achieved with testing for erythrocyte protoporphyrin (>80 mcg/dL) and then fractionation to metal-free and zinc-protoporphyrin IX. If positive, plasma porphyrin and genetic testing is advised (Mittal and Anderson, 2022).

To date, there is no effective treatment for EPP/XLP that preserve or elevates a patient’s quality of life. Patients are advised to avoid sunlight or fluorescent lights. Windows and topical sunscreen offer little to no protection. This greatly affects educational and employment opportunities as well as burdens caregivers (Mittal and Anderson, 2022). Topical ointments such as dihydroxyacetone and naphthoquinone are ill advised due to their carcinogenic potential (Fusaro and Johnson, 1975; Rice, 1976; Fusaro and Rice, 2005). The use of afamelanotide, a synthetic analog of alpha-MSH, has been shown to decrease photosensitivity in those whose lifestyle requires greater sunlight exposure (Minder, 2010; Langendonk et al., 2015; Luger and Bohm, 2015). Afamelanotide was approved by the FDA in October 2019 and is administered subcutaneously in 16 mg implants monthly (Biba, 2014). Some patients have also reported improvements with the use of beta-carotene, possibly via the quenching of free radicals after sun exposure (Kopcke and Krutmann, 2008). NSAIDS and opioids are the standard for patients experiencing severe pain after acute exposure. However, they offer minimal relief as symptoms are typically systemic and may cause patients to be bedridden. Those who suffer from gallstones as a result of porphyrin accumulation may require cholecystectomy as children (Doss and Frank, 1989). Those with protoporphyric hepatopathy undergo treatments that attempt to reduce plasma protoporphyrin (Wahlin et al., 2007). This is achieved via hemin infusions, (Lamon et al., 1980; Reichheld et al., 1999; Dowman et al., 2011), plasmapheresis (Dellon et al., 2002; Wahlin and Harper, 2007), ursodeoxycholic acid (Gross et al., 1998) or cholestyramine (Stathers, 1966; Ishibashi et al., 1999). Ultimately liver transplant may be necessary for those who develop cirrhosis.

## 5 Ferrochelatase in Protozoal Parasites

**FIGURE 7:**
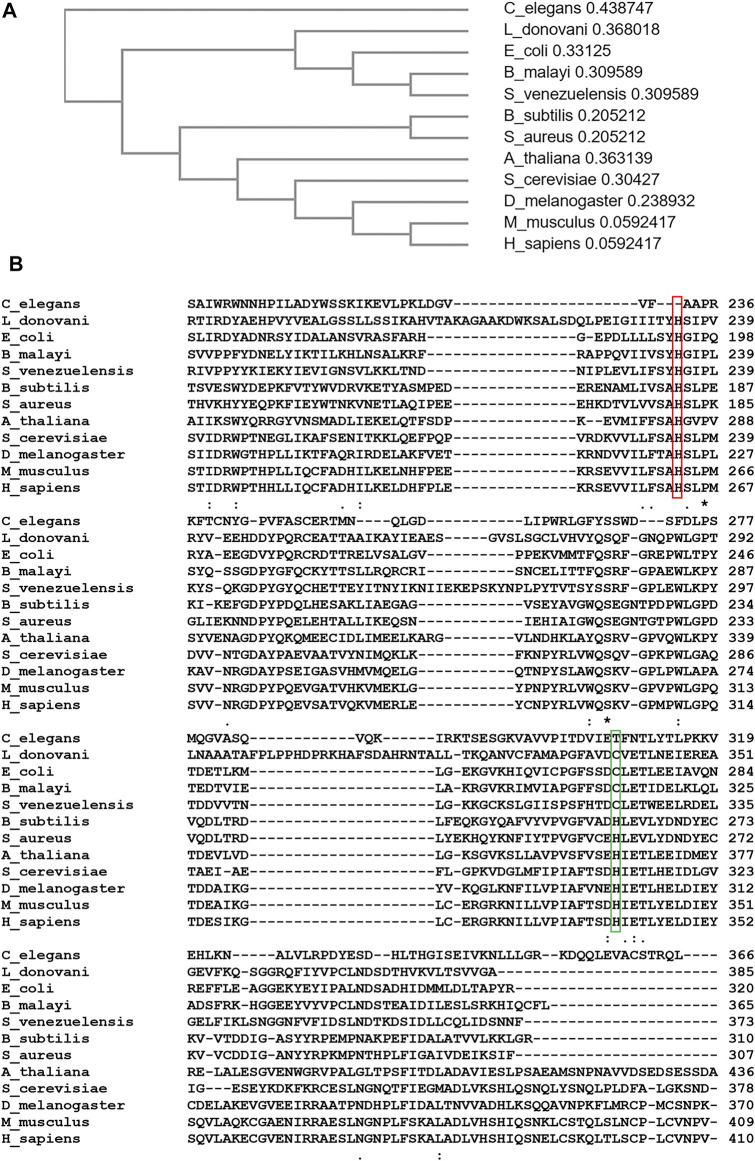
Sequence Alignment and Phylogenic Analyses of Ferrochelatase. **(A)** Cladogram of ferrochelatase from *Caenorhabditis elegans*, *Leishmania donovani*, *Escherichia coli*, *Brugia malayi*, *Strongyloides venezuelensis*, *Bacillus subtilis*, *Staphylococcus aureus*, *Arabidopsis thaliana*, *Saccharomyces cerevisiae*, *Drosophila melanogaster*, *Mus musculus*, and *Homo sapiens*. Value after each organism represents branch length. **(B)** Aligned amino acid sequence of ferrochelatase from above listed organisms. Conserved “catalytic histidine” is highlighted in red. Cysteine residue conserved in ferrochelatases acquired from a gamma-proteobacterium is highlighted in green. **(A,B)** were created using Clustal Omega (Madeira et al., 2019).

Heme is essential for the survival of most organisms including protozoal parasites. Heme auxotrophic parasites such as *Trichomonas vaginalis*, *Entamoeba histolytica* and *Caenorhabditis elegans* cannot synthesize heme since they lack all genes necessary for *de novo* heme biosynthesis (Rao et al., 2005; Koreny et al., 2010). Some parasites such as *Meloidogyne paranaensis*, *Ancylostoma caninum* and *Strongyloides spp* at best possess one heme biosynthetic enzyme (Nagayasu et al., 2013), but are still not sufficient to drive the heme biosynthesis pathway fully or partially. Therefore, organisms in this category primarily obtain heme from their environment or host. In the phylum *Nematoda*, most organisms are heme autotrophs even though some possess a *Fech* gene in their genome. The evolutionary origin of their ferrochelatase has been proposed to be from an alpha-proteobacterium (Nagayasu et al., 2013), which is evolutionarily distinct from ferrochelatase in other non-nematode metazoans. For instance, *Brugia malayi Fech* was suggested to be acquired from obligate endosymbiotic bacteria, *Wolbachia spp*, via lateral gene transfer (Wu et al., 2013). With exception of a ferrochelatase-like gene in *C. elegans* which does not encode a functional enzyme, characterized nematode- ferrochelatases are functionally active (Nagayasu et al., 2013; Wu et al., 2013) and share the same structural features as ferrochelatase in other organisms. In Figure 7, sequence alignment, created using Clustal Omega (Madeira et al., 2019), analysis shows that the ferrochelatase-like gene of *C. elegans* translates to a pseudoferrochelatase, which lacks the essential catalytic histidine (H263 in human) needed for catalysis (Sellers et al., 2001).

In the Trypanosomatidae family, *Leishmania spp* unlike *Trypanosoma* have a partial heme biosynthetic pathway. These genes, proposed to have been acquired from a gamma-proteobacterium, code for enzymes that catalyze the last three steps of this pathway as well as ferrochelatase (Koreny et al., 2010). Thus, *Leishmania spp* can either obtain heme from the host or partially synthesize it from its biosynthetic antepenultimate intermediate, coproporphyrinogen III (Chang and Chang, 1985) depending upon the host and life stage. Following the conversion of coproporphyrinogen III to protoporphyrinogen IX, and then protoporphyrin IX, *Leishmania* ferrochelatase catalyzes the terminal step of heme biosynthesis in the parasite (Orrego et al., 2019). In essence, *Leishmania* ferrochelatase is conditionally essential for their survival.

Due to the essentiality of heme in most protozoal parasitic organisms, inhibiting its synthesis or uptake could impede growth or survival. From phylogenic and sequence alignment analyses (Weerth et al., 2021) (Figure 7), protozoal parasite ferrochelatases are more closely related to microbial ferrochelatases than that of their potential hosts. As a result, inhibitors could be designed to specifically inhibit ferrochelatase in these parasites but not their host.

## 6 Future Directions

While significant progress has been made in understanding the catalytic mechanism, protein-protein interactions, and regulation of ferrochelatase, there are still a number of outstanding questions in these and other areas. These questions contemplate on the additional functions of ferrochelatase beyond the mitochondria, the role of the Fe-S cluster in ferrochelatase, and the stoichiometry and composition of the heme metabolon. Addressing these questions will broaden our knowledge on heme metabolism and regulation, as well as provide a platform for advanced investigations on the biological functions of heme.

### 6.1 Cellular Localization

Ferrochelatase in eukaryotic cells is encoded by the nuclear genome, synthesized in the cytosol with mitochondrial targeting sequence and thereafter translocated to the mitochondria (Camadro and Labbe, 1988; Karr and Dailey, 1988). Recently, Lanzafame et al. (Lanzafame et al., 2021) revealed that ferrochelatase can also be localized to the nucleus. Here, ferrochelatase forms a complex with CSA–CSB–RNAP1– RPS15–RPS10 to regulate rRNA synthesis and processing (Lanzafame et al., 2021). This finding upends the conventional assumption that ferrochelatase in eukaryotic cells is only localized to the mitochondria. These observations raise two key questions. First, does the form of ferrochelatase protein present in the nucleus possess a [2Fe-2S] cluster? Second, do non-catalytic ferrochelatase proteins, such as the one found in *C. elegans*, localize to the nucleus to perform a regulatory role? With regard to the first possibility it would be expected that nuclear localized ferrochelatase would not possess a cluster and not be catalytic. This would be not dissimilar from what is found with the iron responsive protein 1/aconitase where the availability of iron causes a shift in the role that a protein plays *in situ* (Zhang et al., 2014). Since heme is known to regulate gene expression via transcription factors (Ogawa et al., 2001; Sun et al., 2004; Suzuki et al., 2004; Mense and Zhang, 2006), investigating other biological functions of ferrochelatase in the nucleus is worthwhile. For a start, a revisit to ferrochelatase co-IP datasets or further co-IP analysis to identify other nuclear proteins in its interactome would be beneficial.

Moreover, the above-mentioned report on ferrochelatase function within the nucleus (Lanzafame et al., 2021) elicits more questions on heme metabolism 1) do heme levels affect ferrochelatase rRNA regulatory activities?; 2) can ferrochelatase synthesize heme in the nucleus?; and 3) if so, how is protoporphyrin IX trafficked across the mitochondrial membrane to the nucleus? The possibility that the latter events occur stems from a study on ferrochelatase localization in *S. cerevisiae* (Prasad and Dailey, 1995) where it was demonstrated that when ferrochelatase in *S. cerevisiae* is not localized to the mitochondria matrix, synthesis of heme is diminished and the cytochrome content varies. Cells with mislocalized ferrochelatase in this study were still able to synthesize heme although with a significantly lower amount. This phenomenon suggests that protoporphyrin IX was transported from the mitochondria to the mislocalized ferrochelatase for heme synthesis. Thus, a mitochondrial protoporphyrin IX transporter(s) likely exists. In addressing these questions and speculations, factors that drive this hypothetical protoporphyrin IX transport from the mitochondria could be identified, alongside non-conventional mechanisms for heme metabolism and regulation.

### 6.2 Role of the Fe-S Cluster

Fe–S clusters are ancient, biological prosthetic groups (Beinert, 2000). Their primordial function, electron transport (Hudson et al., 2005; Brzoska et al., 2006) has been persevered from the anoxic, anaerobic era to this modern, oxygen-rich era. In the mitochondria and chloroplast of eukaryotic cells, Fe-S clusters play crucial roles in biological processes that depend on electron transfer such as, cellular respiration and photosynthesis, respectively (Vassiliev et al., 2001; Stehling and Lill, 2013; Stiban et al., 2016; Ibrahim et al., 2020). Moreover, a significant number of proteins not directly involved in these biological processes or in other organelles also have these clusters. With the focus on metazoan ferrochelatases, the Fe-S cluster, which has a low mid-point potential of −450 mV (Burden et al., 1999), is crucial for stability and function (Dailey et al., 1994a; Sellers et al., 1996; Crooks et al., 2010). A recent report also describes a correlation between Fe-S clusters in ferrochelatase and aerobic metabolism (Weerth et al., 2021). So, to be evolutionarily conserved with preference to aerobic organisms, Fe-S clusters in ferrochelatase may have a significant biological function beyond conferring structural stability.

Aerobic organisms utilize oxygen for cellular respiration and consequently generate ROS as by-products. Organisms in this category are thereby exposed to the detrimental effects of oxidative stress caused by ROS. To mitigate these effects, these organisms can sense elevated levels of ROS through Fe-S clusters-containing proteins and other molecular machineries and initiate response to this stimulus by regulating the expression of ROS-associated genes (Dietz, 2014; Crack and Le Brun, 2018; Rouault, 2019; Mariotti et al., 2020). Based on the outlined conditions that; 1) Fe-S clusters in metazoan ferrochelatases are sensitive to pH and redox potential (ROS) (Sellers et al., 1996; Shah et al., 2012), and 2) ferrochelatase forms a complex with other nuclear proteins to regulate rRNA transcription (Lanzafame et al., 2021), it is possible that ferrochelatase might also act as a redox sensor for signal transduction and gene regulation. To this end, if Fe-S clusters are present in ferrochelatase localized to the nucleus, then, their role in regulating gene expression needs to be explored.

### 6.3 Metabolon

The mitochondrial ferrochelatase metabolon has been well studied (Medlock et al., 2015; Piel et al., 2019; Dietz et al., 2021), however, neither the stoichiometry of this complex, nor the differences in the metabolon in different cell types and developmental stages has been fully determined. It is necessary to understand how the expression levels of participating proteins could affect the integrity of the complex and consequently the metabolism of heme. Biochemical and biophysical approaches such as CryoEM, X-ray crystallography, and small angle X-ray scattering (SAX) have been adopted in protein complex stoichiometry studies (Forster et al., 2008; Okamura-Ikeda et al., 2010; Bai et al., 2015; Boniecki et al., 2017; Costa et al., 2017; Schneidman-Duhovny and Hammel, 2018; Fox et al., 2019). Utilizing one or a combination of these techniques would be helpful in elucidating the structural foundation of the ferrochelatase interactome. Proximity labeling (De Munter et al., 2017; Qin et al., 2021) and NMR (Liu et al., 2016) could also be employed to study crucial transient interactions. In characterizing the stoichiometry of ferrochelatase metabolon, interaction interfaces of participating proteins would be identified. This may provide answers as to why mutations in non-heme biosynthetic enzymes in mitochondrial heme metabolon affect heme metabolism.

## 7 Conclusion

Over the past 20 years, significant progress has been made in understanding the intersection of iron and porphyrin metabolism which occurs at the enzyme ferrochelatase. Structural studies of human ferrochelatase, both the wild-type and variants with different substrates, have provided new information on the catalytic mechanism and impetus to better understand ferrochelatase protein partners. Studies to identify these partners led to the discovery of the mitochondrial heme metabolon, which delineates how substrates are channeled to ferrochelatase for heme synthesis and heme is subsequently transported to other mitochondrial compartments. Recent studies on this metabolon will likely illustrate the physiologic functions of these non-heme biosynthetic enzymes in the complex as indispensable for the metabolic process since they play key regulatory roles. On the part of ferrochelatase regulation, novel post translational modifications discussed here also provide a better understanding to the dynamic regulation of heme homeostasis via ferrochelatase in response to different physiological conditions. It is of note that regulation of ferrochelatase activity has proven in recent years to be imperative, as several researchers are exploring the aspects of ferrochelatase inhibition with small-molecule inhibitors for therapeutic purposes in the treatment of cancers, ocular angiogenesis, and parasitic infections. With the present knowledge and understanding of iron and porphyrin metabolism intersection at ferrochelatase, there are multiple novel avenues to investigate in order to better understand the role of iron, porphyrin and heme homeostasis in human health and disease.
