# Supplementary material for: Ferrochelatase: Mapping the Intersection of Iron and Porphyrin Metabolism in the Mitochondria
Source: Front Cell Dev Biol. 2022 May 12;10:894591. doi: 10.3389/fcell.2022.894591 (PMC9133952; doi:10.3389/fcell.2022.894591)
Supplement: Supplementary file 1 [file Table1.DOCX]

**Supplemental Table 1. Summary of published protoporphyrin ferrochelatase structures**

| **PDB ID** | **Species** | **Description** | **Resolution (Å)** |
| --- | --- | --- | --- |
| 2QD3 | Human | Wild-type | 2.2 |
| 2QD4 | Human | Wild-type | 2.0 |
| 3HCO | Human | Wild-type with Cd protoporphyrin | 1.8 |
| 3HCN | Human | Wild-type with Hg protoporphyrin | 1.6 |
| 3HCP | Human | Wild-type with Mn deuteroporphyrin | 2.0 |
| 3HCR | Human | Wild-type with Ni deuteroporphyrin | 2.2 |
| 2QD5 | Human | Wild-type with Pb protoporphyrin | 2.3 |
| 3W1W | Human | Wild-type with salicylic acid | 2.0 |
| 7CTC | Human | Wild-type with inhibitor complex 1 | 2.0 |
| 7CT7 | Human | Wild-type with inhibitor complex 2 | 2.0 |
| 4KMM | Human | M76H with heme bound partial occupancy | 2.5 |
| 2QD2 | Human | F110A with heme bound | 2.2 |
| 4MK4 | Human | F110A/S197C | 2.5 |
| 2HRC | Human | R115L | 1.7 |
| 1HRK | Human | R115L | 2.0 |
| 3AQI | Human | H240A | 1.7 |
| 2PO5 | Human | H263C/R115L | 2.2 |
| 2PO7 | Human | H341C/R115L | 2.2 |
| 4KLA | Human | E343D with protoporphyrin partial occupancy | 2.6 |
| 4KLC | Human | E343D/F110A with heme partial occupancy | 2.4 |
| 2QD1 | Human | E343K with protoporphyrin | 2.2 |
| 2HRE | Human | E343K with protoporphyrin | 2.5 |
| 4KLR | Human | E343Q with metalated protoporphyrin | 2.1 |
| 2PNJ | Human | F337A/R115L | 2.3 |
| 4F4D | Human | F337R | 1.8 |
| 1LBQ | *S. cerevisiae* | Wild-type | 2.4 |
| 1L8X | *S. cerevisiae* | Wild-type with Co | 2.7 |
| 7L78 | *S. cerevisiae* | H235C | 2.4 |
