# Supplementary material for: Ferrochelatase: Mapping the Intersection of Iron and Porphyrin Metabolism in the Mitochondria
Source: Front Cell Dev Biol. 2022 May 12;10:894591. doi: 10.3389/fcell.2022.894591 (PMC9133952; doi:10.3389/fcell.2022.894591)
Supplement: Supplementary file 2 [file Table2.DOCX]

**Supplemental Table 2. Summary of published coproporphyrin ferrochelatase structures**

| **PDB ID** | **Species** | **Description** | **Resolution (Å)** |
| --- | --- | --- | --- |
| 1DOZ | *B. subtilis* | Wild-type | 1.8 |
| 1AK1 | *B. subtilis* | Wild-type | 1.9 |
| 3M4Z | *B. subtilis* | Wild-type with Co | 1.9 |
| 1N0I | *B. subtilis* | Wild-type with Cd | 2.0 |
| 2HK6 | *B. subtilis* | Wild-type with Fe | 1.7 |
| 1LD3 | *B. subtilis* | Wild-type with Zn | 2.6 |
| 1C1H | *B. subtilis* | Wild-type with *N*-methylmesoporphyrin | 1.9 |
| 1C9E | *B. subtilis* | Wild-type with Cu *N*-methylmesoporphyrin | 2.3 |
| 2Q2N | *B. subtilis* | Wild-type with 2,4-disulfonic deuteroporphyrin IX | 1.8 |
| 2AC2 | *B. subtilis* | Y13F with Zn | 2.5 |
| 3GOQ | *B. subtilis* | Y13M | 1.6 |
| 2H1V | *B. subtilis* | K87A | 1.2 |
| 2H1W | *B. subtilis* | H183A | 2.6 |
| 2Q3J | *B. subtilis* | H183A with *N*-methylmesoporphyrin | 2.4 |
| 2AC4 | *B. subtilis* | H183C | 2.1 |
| 2Q2O | *B. subtilis* | H183C with 2,4-disulfonic deuteroporphyrin IX | 2.1 |
| 2C8J | *B. anthracis* | Wild-type | 2.1 |
| 6RWV | *L. monocytogenes* | Wild-type | 1.6 |
| 6SV3 | *L. monocytogenes* | Wild-type with coproheme | 1.6 |
